# DomAda-FruitDet: Domain-Adaptive Anchor-Free Fruit Detection Model for Auto Labeling

**DOI:** 10.34133/plantphenomics.0135

**Published:** 2024-01-22

**Authors:** Wenli Zhang, Chao Zheng, Chenhuizi Wang, Wei Guo

**Affiliations:** ^1^ Information Department, Beijing University of Technology, Beijing 100022, China.; ^2^ Graduate School of Agricultural and Life Sciences, The University of Tokyo, Tokyo 188-0002, Japan.

## Abstract

Recently, deep learning-based fruit detection applications have been widely used in the modern fruit industry; however, the training data labeling process remains a time-consuming and labor-intensive process. Auto labeling can provide a convenient and efficient data source for constructing smart orchards based on deep-learning technology. In our previous study, based on a labeled source domain fruit dataset, we used a generative adversarial network and a fruit detection model to achieve auto labeling of unlabeled target domain fruit images. However, since the current method uses one species source domain fruit to label multiple species target domain fruits, there is a problem of the domain gap in both the foreground and the background between the training data (retaining the source domain fruit label information) and the application data (target domain fruit images) of the fruit detection model. Therefore, we propose a domain-adaptive anchor-free fruit detection model, DomAda-FruitDet, and apply it to the previously proposed fruit labeling method to further improve the accuracy. It consists of 2 design aspects: (a) With a foreground domain-adaptive structure based on double prediction layers, an anchor-free method with multiscale detection capability is constructed to generate adaptive bounding boxes that overcome the foreground domain gap; (b) with a background domain-adaptive strategy based on sample allocation, we enhance the ability of the model to extract foreground object features to overcome the background domain gap. As a result, the proposed method can label actual apple, tomato, pitaya, and mango datasets, with an average precision of 90.9%, 90.8%, 88.3%, and 94.0%, respectively. In conclusion, the proposed DomAda-FruitDet effectively addressed the problem of the domain gap and improved effective auto labeling for fruit detection tasks.

## Infographic

**Figure:**
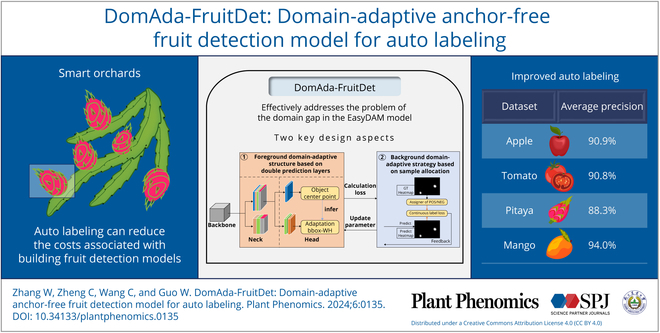

## Introduction

Deep learning-based fruit detection, combined with agricultural machinery, can be applied to many intelligent tasks in smart orchards, such as fruit positioning, fruit yield prediction, and automatic fruit picking [1–6]. However, training a fruit detection model relies on many labeled datasets [7–9]. Moreover, due to the poor generalization performance of the model, it is always requested to label new image data and build a new model that can be adapted to new tasks, which is time-consuming, laborious, and becomes a bottleneck of deep learning-based applications [10]. Thus, automatic labeling is receiving increasing attention; for instance, a fruit image auto labeling work can automatically generate bounding boxes as the labels without manual operation.

In our previous research [11,12], EasyDAM was proposed to accomplish fruit auto labeling. Based on a labeled source domain fruit dataset, this method can automatically label various species of unlabeled target domain fruit images with high accuracy, thus saving labor costs. The main steps are as follows: First, based on the labeled source domain fruit images, the synthetic fruit images of the target domain are obtained through a GAN (generative adversarial network). Then, the labeled target domain synthetic fruit images are fed into Orange-YOLO [13] (a detection model proposed by our team early on) as training data. Finally, the unlabeled target domain actual fruit images are input to the pretrained fruit detection model to obtain pseudo-label, which can be converted into image label data.

**Fig. 1.**
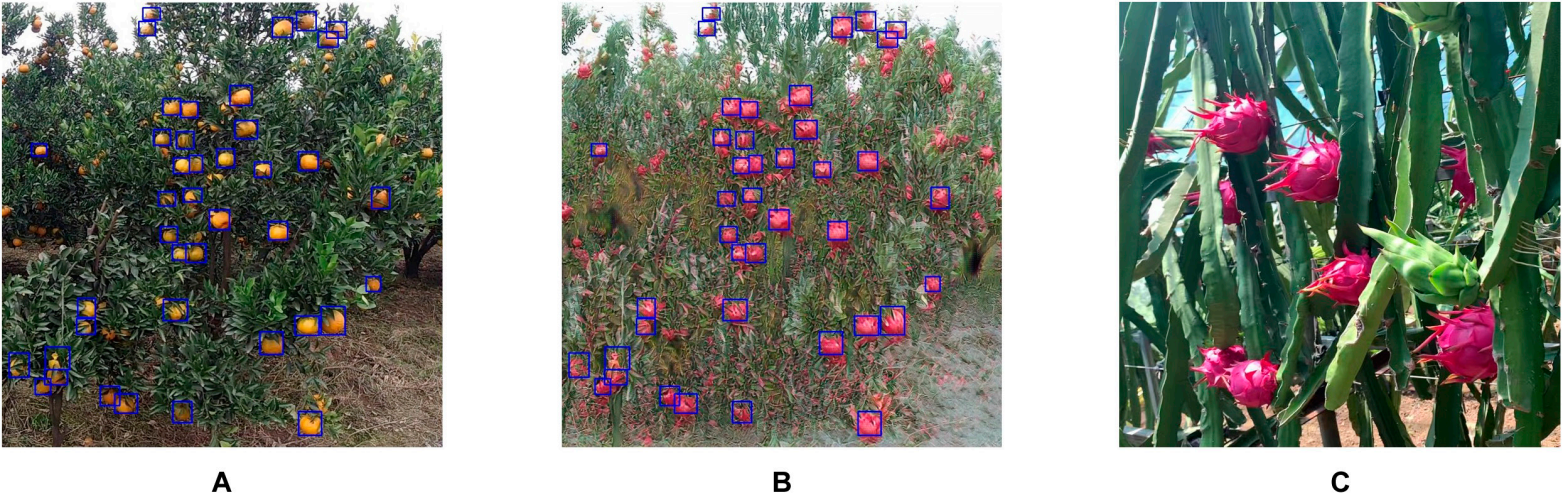
Illustration of the domain gap of the EasyDAM method, with orange as the source domain and pitaya as the target domain, for example (the blue rectangular boxes in the images represent the labeled information). (A) The source domain orange image, (B) the target domain synthetic pitaya image (training data of the fruit detection model), and (C) the target domain actual pitaya image (testing data of the fruit detection model), where (B) is generated from (A) by the GAN, thus causing a substantial domain gap between (B) and (C) in both the foreground and the background.

The problem where models, trained on one particular dataset (also known as source dataset), do not generalize well to a dataset that has a different distribution (also known as target dataset) is commonly referred to as domain gap or distribution gap [14]. However, in the EasyDAM process, the training data of the fruit detection model are synthetic fruit images of the target domain (generated from the source domain fruit images by the GAN), while the testing data (to be labeled) of the fruit detection model are actual fruit images of the target domain in the orchard scene, as shown in Fig. 1. This leads to a large domain gap between the training data and the actual testing data of the fruit detection model, and the ability of the model to bridge this domain gap directly affects the auto labeling accuracy. Specifically, the domain gap can be refined into 2 aspects, the foreground domain gap and the background domain gap:

1. The foreground domain gap (e.g., in Fig. 1B, where the fruit labeled box does not match the fruit object scale in Fig. 1C): Because the foreground objects of various target domain synthetic fruit images (as in Fig. 1B) are generated from the foreground of the source domain fruit images (as in Fig. 1A) by a GAN, their supervisory label information still retains the scale characteristics of the source domain fruits. However, due to the different shooting angles, shooting distances, and species of the target domain fruits, the scale characteristics of the actual fruit in the target domain (as shown in Fig. 1C) are diverse. As a result, there is a substantial difference in the scale characteristics between the synthetic fruit and the actual fruit, thus causing the foreground domain gap problem. This causes the quality of the pseudo-label generated by the fruit detection model to be affected.

2. The background domain gap (e.g., in Fig. 1B and C, where there is a substantial difference in image background between them): Because the target domain synthetic fruit images (as in Fig. 1B) are generated from the source domain fruit images (as in Fig. 1A), only the foreground object of the fruit is converted, and its background remains the background of the source domain fruit images. However, the target domain actual fruit images (as in Fig. 1C) are the background of the natural scene of the fruit, which causes the background domain gap problem and further affects the quality of the pseudo-label generated by the fruit detection model.

The current adaptive object detection in the dominant field can be classified into 3 categories according to the implementation methods [14]: (a) adversarial feature learning, (b) image-to-image translation, and (c) pseudo-label-based self-training. Among them, adversarial feature learning [15,16] utilizes a classifier to correctly classify the features extracted by the detector into the source/target domain, while the detector is trained to deceive the classifier. When the classifier fails to categorize correctly, it indicates that the detector can extract domain-invariant features. This method has the ability for domain adaption, but it needs to extract the target domain features in advance, which cannot satisfy the situation when the actual scene data is unknown, so it is not applicable to EasyDAM. In addition, image-to-image translation [17,18] generates the intermediate domain data between the source and target domains through image transformation and retrains the model. However, the direction of generation is usually a style conversion, such as sunny to foggy and day to night; thus, it cannot solve the domain gap of EasyDAM. Pseudo-label-based self-training [19,20] feeds the target domain images into the pretrained model to obtain pseudo-labels, thus updating the model to enhance the detection capability. However, the available pseudo-labels rely on a pretrained model with some target domain detection ability. When the pretrained model is not generalized enough, the pseudo-labels will mislead the model to perform worse. It can be seen that the current dominant methods focus on how to import the target domain features to train the model while neglecting the development of the generalization ability of the model structural design. Therefore, this paper attempts to address the domain gap of the EasyDAM method from the perspective of detection model design.

To address the above problems, we further analyze the related research of the object detection model from 2 aspects:

*1. Related research on the scale feature of foreground objects:* At present, most researchers [21–24] adopt anchor-based models for fruit detection, such as apples [21], mangoes [22], peaches [23], and pears [24]. This kind of detector introduces prior knowledge of fruit object scale features through the setting of anchor boxes to adapt to generate bounding boxes of fruit objects. In addition, some researchers [25–27] have further designed multiscale structures to increase the accuracy of detection under various scales. The above methods are mainly based on the idea that the training data and the actual testing data obey the same scale information distribution to train the model, ensuring the detection performance of the testing data. However, in the case of different scale characteristics of fruits between the source domain and the target domain, the anchor box parameters in such detectors (adopted by the original EasyDAM method) mainly come from the fruit scale feature in the source domain, which is difficult to effectively apply to the target domain fruit with various scale condition scenes.

Therefore, some researchers [28–30] have begun to design anchor-free detectors. This kind of model does not need to preset the anchor boxes. It is not limited to the influence of the object scale feature in the training data during prediction to avoid the shortcoming of the lack of flexibility in the generation of bounding boxes. Several scholars [31–34] have already applied the anchor-free detector for fruit detection. Liu et al. [31] proposed TomatoDet for tomato detection, which avoids the complex hyperparameter design and low detection efficiency caused by exhaustive anchor boxes and classification operations in anchor-based detectors. However, the circular bounding boxes generated by this model are only suitable for tomatoes or other round fruits with aspect ratios close to 1:1, not for other species with substantial differences in aspect. Ji et al. [32] proposed ShufflenetV2-YOLOX for apple detection, which avoids the computational burden caused by anchor boxes, thus achieving a balance between speed and accuracy. However, this model is designed specifically for the apple feature and cannot be effectively applied to other species. Wei et al. [33] proposed D2D to detect green persimmon and green apple, which avoids a large amount of computation and storage resources by designing an anchor-free structure. However, it performs poorly for fruits with colors other than green. Zhao and Yan [34] used CenterNet to detect 4 species of fruits: apple, banana, orange, and pear. Their method avoids the drawbacks of anchor-based detectors, such as complex parameter tuning and high computational costs. However, this method is designed for detecting fruits placed on a table after picking and objects with a relatively large scale in the image. It is challenging to apply it in complex natural orchards.

The recent research on fruit detection by using anchor-free detectors focuses on achieving a balance between speed and accuracy by avoiding the hyperparameters and computational effort associated with anchor boxes. Meanwhile, the models designed in such studies perform well only for single fruit species or a fixed shooting method and scenario. However, it is not easy to generalize and adapt to fruit detection tasks with different species and multiple-scale characteristics in complex natural orchards. Therefore, the primary focus of this study is to leverage the anchor-free characteristic, which is not limited by prior anchor box scale information, to develop a structure that can manage the diverse scales of various target domain fruits in the actual orchard. This approach aims to address the problem of the scale domain gap in detecting fruit foreground objects.

*2. Related research on the learning capability and learning approach of foreground–background feature in the image:* Recently, there are studies on how to avoid interference from the background and to improve the ability of detectors to extract foreground features. To address vehicle and pedestrian detection tasks in different street scenes, some scholars [35–37] have added classifiers during model training to extract domain-invariant features between training data and testing data, thus improving the ability of the model to extract foreground object features under different backgrounds. However, this approach requires prior knowledge about the foreground–background data distribution in the scenario to train the detectors, which is inconvenient for practical applications. In addition, in the general detection model design, some researchers [38–40] have adjusted the learning ability of the model to foreground–background features by designing positive and negative sample allocation strategies. Kim and Lee [38] proposed the PAA method, which uses the confidence scores and the Intersection over Union (IoU) between the anchor box and the Ground Truth (GT) box to calculate the score of this anchor box. This method establishes a probability distribution function based on the scores of all anchor boxes to determine the positive and the negative samples, thus avoiding the insufficient feature learning of the foreground in the complex background caused by using only the IoU to define samples. Zhang et al. [39] proposed the Adaptive Training Sample Selection method, which can automatically allocate positive and negative samples based on adaptive dataset characteristics. It enables the detector to fully learn both the foreground and background features of the training data. Ge et al. [40] proposed the Optimal Transport Assignment method, which uses a “supply-demand” relationship for global pairing, enabling each GT box to obtain optimal positive and negative samples from a global perspective for training. Their method allows the optimal path for the foreground and background feature learning approach, resulting in better performance.

As seen, positive and negative sample allocation strategies of detectors can adjust the focus and extraction ability of the foreground–background feature in the model. However, the recent research focuses on balancing positive and negative samples to ensure that the model can fully learn both foreground and background features and cannot make the model more focused on foreground features while reducing the influence of complicated backgrounds. Therefore, designing a positive and negative sample allocation strategy for the model to enhance the extraction ability of fruit features and to weaken the interference between different backgrounds of training and testing data provides a potential solution to address the problem of the background domain gap.

In summary, to address the problem of the domain gap between training and testing data in the fruit detection model of EasyDAM [11,12], in this paper, we propose a new domain-adaptive anchor-free fruit detection model called DomAda-FruitDet to further improve the accuracy of fruit labeling. The main contributions of this paper are as follows:

1. Different from domain-adaptive object detection methods that require additional application data to be imported to train the model, DomAda-FruitDet is designed from the perspective of detection model structure. It can generalize the detection capability obtained from the training data features to the actual application data, and thus adaptively generate detection results for the target domain fruit dataset.

2. In addition, to address the problem of the scale domain gap of fruit foreground objects and the different backgrounds between the training and testing data, DomAda-FruitDet proposes a foreground domain-adaptive structure and a background domain-adaptive strategy. The method can effectively bridge the domain gap between the single source domain fruit and the diverse target domain fruits.

## Methods

**Fig. 2.**
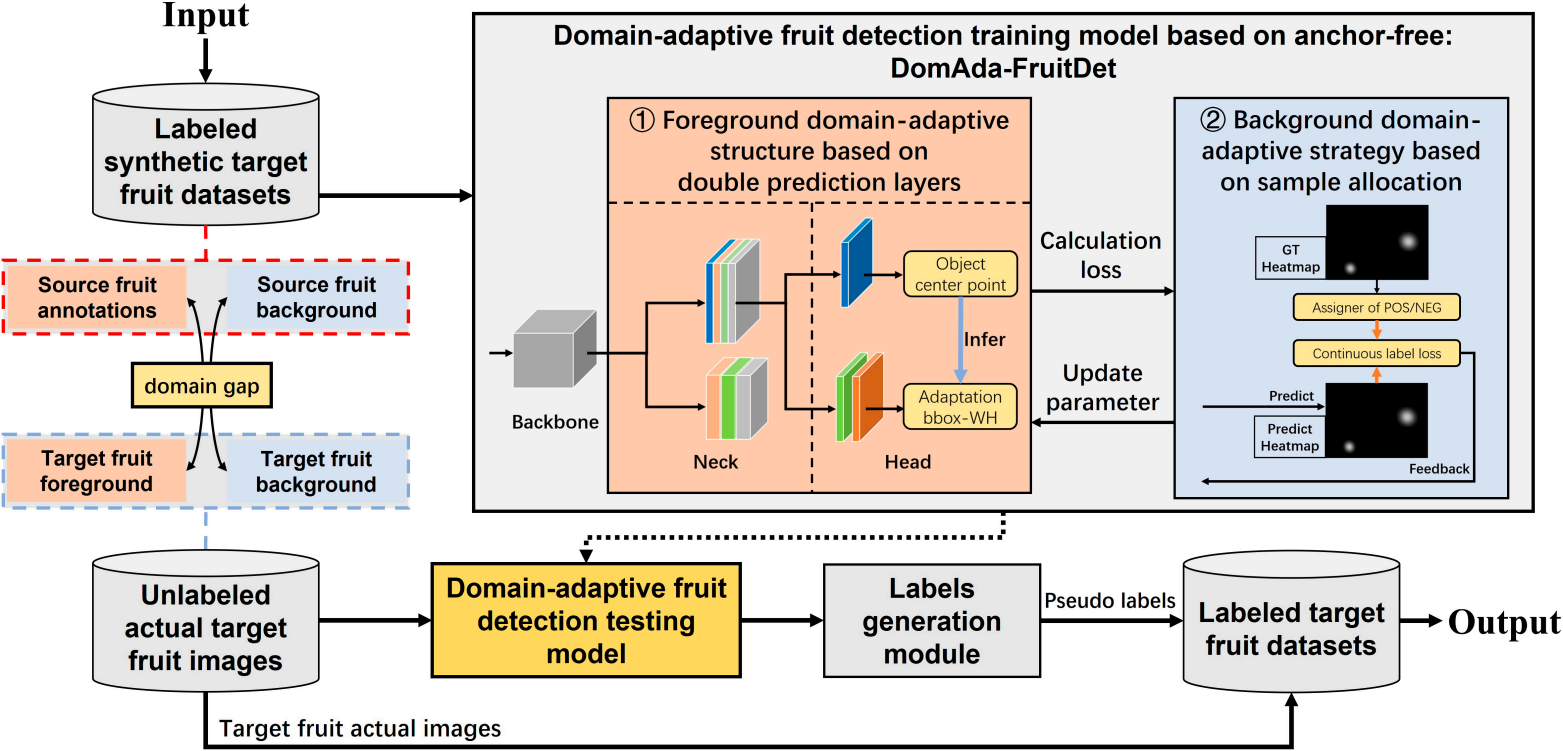
Flowchart for using DomAda-FruitDet to improve the accuracy of the EasyDAM method, with the labeled target domain synthetic fruit dataset as input and the obtained labeled target domain actual fruit dataset as output. The model designed in this paper includes 2 improvement points: (a) By using the foreground domain-adaptive structure based on double prediction layers in ①, the method of generating bounding boxes by locating the center point and for predicting fruits of different scales by different layers can effectively address the problem of the foreground domain gap; (b) by using the background domain-adaptive strategy based on sample allocation in ②, the method of enhancing the ability of the model to extract foreground features can effectively address the problem of the background domain gap.

In this paper, we propose a domain-adaptive fruit detection model for the EasyDAM [11,12] method to improve the accuracy of fruit data labeling, and the overall framework is shown in Fig. 2.

To address the problem of the domain gap, we choose CenterNet [29], which is anchor-free, as the baseline model for designing the domain-adaptive fruit detection model (as shown in Fig. 2) developed in this paper, named DomAda-FruitDet. With DomAda-FruitDet’s capability, we can achieve high-precision labeling of the target domain fruit dataset.

DomAda-FruitDet has 2 key design points: For the foreground domain gap, we design a foreground domain-adaptive structure based on double prediction layers (as shown in Fig. 2, ①, introduced in the “Foreground domain-adaptive structure based on double prediction layers” section). By locating the object’s center point and by detecting objects of different scales in different layers, the method can effectively adapt to the scale of the fruit to generate bounding boxes. Additionally, for the background domain gap, we further design a background domain-adaptive strategy based on sample allocation (as shown in Fig. 2, ②, introduced in the “Background domain-adaptive strategy based on sample allocation” section). By enhancing the ability of the model to extract foreground object features, the method can effectively avoid the influence of different background scenes on fruit detection.

### Foreground domain-adaptive structure based on double prediction layers

**Fig. 3.**
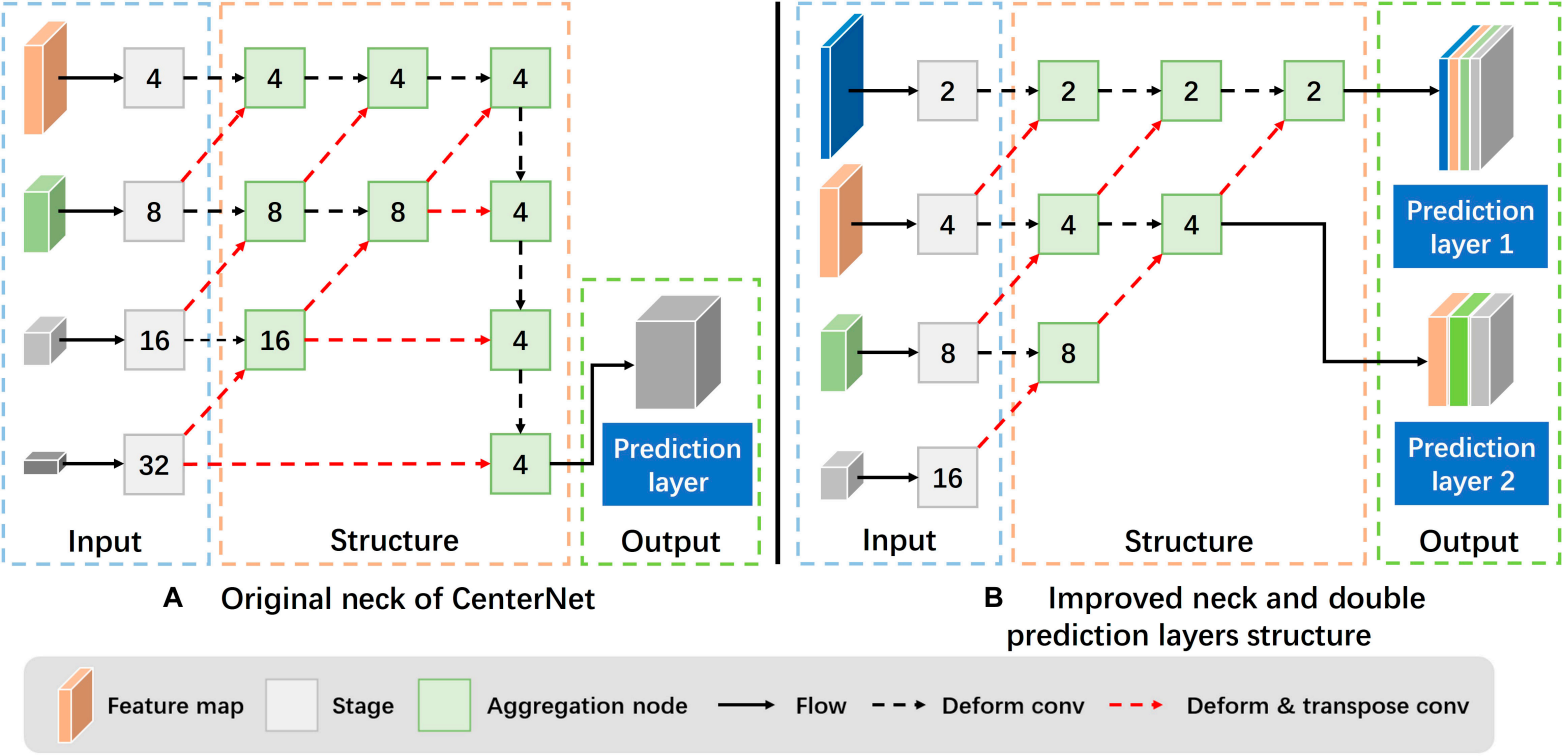
(A) The neck of CenterNet outputs a prediction feature map for object detection at each scale only. (B) The improved foreground domain-adaptive structure based on double prediction layers outputs 2 prediction feature maps for detecting fruit objects of various scales. The numbers in the figure represent the downsampling factor of this feature map. Three parts are improved separately: the input of the neck, the internal feature fusion structure of the neck, and the prediction layer output of the neck.

The original CenterNet utilizes the anchor-free detection principle based on the center key point, which can generate bounding boxes that are not limited by the scale characteristics of training data to some extent. However, its neck has only a single prediction layer, making it difficult to detect objects of various scales accurately, and CenterNet cannot resolve the issue of the foreground domain gap. Therefore, to achieve foreground scale domain adaptation, we comprehensively analyzed the scale characteristics of fruit objects under the domain gap and improved the neck of the original model accordingly, and we refer to it as the foreground domain-adaptive structure based on double prediction layers. This structure mainly includes 2 design components called the double prediction layer design for fruit scale characteristics and the cropping design for fruit scale feature generalization. The comparison between the original and improved neck is shown in Fig. 3.

**Fig. 4.**
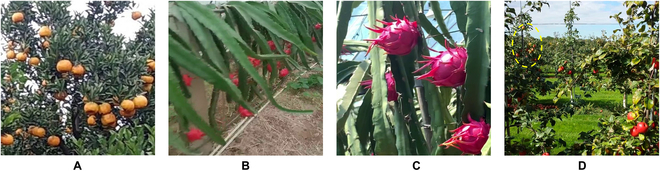
Example of the scale characteristics of source domain orange and target domain fruits. (A) Orange scale characteristics: Each orange has a similar scale. (B and C) Target domain pitaya scale characteristics: Different scenes and shooting methods can lead to substantial differences in the scale of the same species. Compared with (A) and (D), the scale of different species also has a substantial difference. (D) Target domain apple scale characteristics: As shown in the yellow circle, there are some extremely small objects.

*1. The double prediction layer design for fruit scale characteristics:* After analyzing the dataset used and the definition criteria for object scales, we know that different species of target domain fruits present different scale characteristics in the image. The fruit scale range in all target domain fruit images is extensive and substantially different from the source domain fruit. At the same time, there are some small fruits (pixel area < 32 × 32 or the square root of the relative area ratio <3%) in the image, which have a crucial impact on the detection accuracy due to the fact that fewer features that can be extracted, as shown in Fig. 4.

According to the comprehensive analysis of the relationship between the depth of the convolution layer, the resolution of the feature map, and the scale of the object to be detected by researchers [41], we know that the shallow feature map has a smaller receptive field, focusing on the color and texture details of the image and that it is more suitable for detecting smaller objects. In comparison, the deep feature map has a larger receptive field, contains a rich semantic feature, and is more suitable for detecting larger objects. Therefore, based on the above analysis and principles, to build the multiscale detection ability and to detect small fruits accurately, we modify the input of the neck and design 2 prediction layers’ output for detecting objects with various scales, as described below.

1. The input of the neck: As shown in the comparison between the *Input* in Fig. 3A and B, a feature map from the shallower layer of the backbone is added while deleting the feature map from the deepest layer. In this way, the shallower feature map can fully retain the fruit detail information and can avoid interference from an overly deep feature map. Meanwhile, the prediction feature map output by the neck can be obtained based on the feature maps with the same downsampling factor and does not suffer from feature loss issues caused by upsampling.

2. The prediction layers (the output of the neck): As shown in Fig. 3B, *Output*, Prediction Layer No. 1 outputs a 2× downsampling prediction feature map to detect the smaller fruits. This feature map is fused from different layers of the backbone, ensuring the detection ability of small fruit objects with rich detail features based on high-resolution characteristics while retaining the fruit semantic information from the deeper layers. At the same time, because the fruit semantic feature is relatively simple and does not require a deep prediction layer to locate large-scale fruit objects in the orchard, Prediction Layer No. 2 outputs a 4× downsampling prediction feature map to detect the fruits at other scales. This feature map is fused from all layers of the backbone except for the shallowest layer and has a suitable resolution. The map avoids too much detail feature interference from the shallowest layer and ensures the detection ability of fruits other than small objects.

In the end, based on the fruit scale distribution characteristics applicable to orchards, 2 different resolution prediction feature maps targeted to detect fruits of various scales, effectively addressing the problem of the scale domain gap of fruit foreground objects.

*2. The cropping design for fruit scale feature generalization:* The excessive fusion of deep features during feature fusion in the neck is a crucial reason for overfitting and reducing the generalizability of the model. The training data of the fruit detection model are generated from the same source domain fruit, which has a similar scale distribution. However, the actual testing data have a variety of fruit scales, so it is necessary to avoid any operations that can affect the generalization in neck design for this model.

However, the feature fusion method in the original neck of CenterNet includes 2 parts: fusion from deep to shallow and fusion from shallow to deep, with the latter leading to the problem of excessive fusion of deep features. Therefore, we crop the fusion from shallow to deep while retaining the fusion from deep to shallow with the iterative aggregation structure; this allows the model to construct the neck and the prediction feature map, as shown in *Structure* (A) and (B) in Fig. 3. In the original structure, the prediction feature map is output from the fusion from shallow to deep part, as shown in Eq. 1.

Fnm=4=DCFn−1m=4+DUOm=2n+2n=1,2,3 (1)

In Eq. 1, *F*, *n*, *m*, *DC*, *DU*, and *O* represent the feature map from the fusion from the shallow to the deep part, the number of times the feature map is fused in the fusion from the shallow to the deep part (the prediction feature map is output when *n* = 3), the downsampling factor of the feature map, deformable convolutional operation [29], deformable convolutional and upsampling operation [29], and the feature map output from the fusion from the deep to the shallow part, respectively. As indicated by Eq. 1, in the process of fusing and outputting the prediction feature map from shallow to deep, each fusion operation introduces an even deeper feature map. The gradual fusion leads to a decreasing proportion of information contained by shallow feature maps in the final output prediction feature map. As a result, too many deep features cause the model to focus excessively on specific shapes and contours of the objects while ignoring the diversity of fruits in different perspectives, sizes, and postures. In the cropped neck, the prediction feature map is output from the fusion from the deep to the shallow part, as shown in Eq. 2.

Onm=DCOn−1m+DUOn−12m (2)

In Eq. 2, *O*, *n*, *m*, *DC*, and *DU* represent the feature map from the fusion from the deep to the shallow part, the number of times the feature map is fused with the same resolution, the downsampling factor of the feature map (*m* = 2, 4, 8, 16; when *m* = 2, *n* = 1, 2, 3; when *m* = 4, *n* = 1, 2; when *m* = 8, *n* = 1; when *m* = 16, *n* = 0. In this paper, the prediction feature map is output when *m* = 2, *n* = 3 and *m* = 4, *n* = 2), deformable convolutional operation, and deformable convolutional and upsampling operation, respectively. As indicated by Eq. 2, after cropping the fusion from the shallow to the deep part, the prediction feature map is directly output from the fusion from the deep to the shallow part. Each fusion operation introduces an even shallower feature map. The final prediction feature map not only retains the rich fruit details contained by shallow feature maps but also avoids interference from the excessive fusion of deep features. In addition, directly outputting from the fusion from the deep to the shallow part enables a more flexible resolution for the prediction feature map, which is not limited to the disadvantage of being output with only 4× downsampling resolution.

Through the above method, the cropped neck avoids operations that affect the generalizability of the model and that maximizes the fruit feature extracted from the backbone to construct the prediction layer, which further ensures the ability of the model to detect multiscale fruits under the domain gap.

In summary, based on CenterNet, we design a foreground domain-adaptive structure based on double prediction layers. By locating fruits with center points and by detecting different scale objects through prediction layers with different resolutions, this structure achieves multiscale fruit detection and adaptive bounding box generation. Our approach can effectively bridge the domain gap of fruit foreground objects through the above method, thereby improving the accuracy of pseudo-label generation.

### Background domain-adaptive strategy based on sample allocation

Foreground domain-invariant features refer to the shared features of foreground objects in different background domains. Making the model focus more on extracting the fruit foreground features is an effective way to achieve cross-background domain detection. However, the original CenterNet suffers from an extremely unbalanced allocation of positive and negative sample labels, with far fewer positive samples used to learn foreground features than negative samples used to learn background features. This makes the model unable to fully extract the foreground object feature, leading to difficulties in addressing the problem of the background domain gap. In this paper, we analyze the original heatmap branch of CenterNet, which is used to learn foreground–background features for object localization, and we develop an improved sample allocation strategy.

**Fig. 5.**
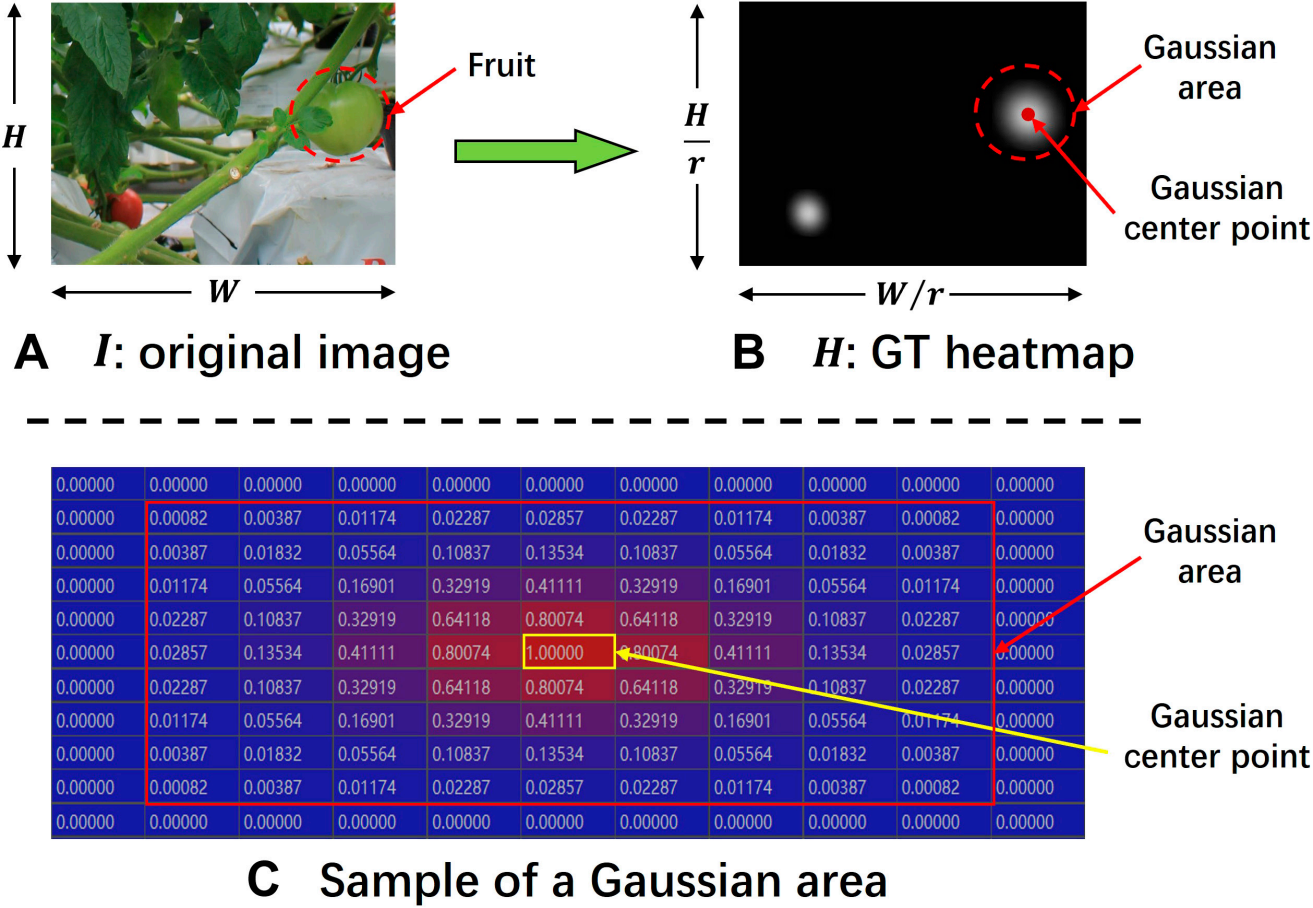
(A) Original fruit image *I*, (B) encoded ground truth heatmap *H*, and (C) sample of Gaussian encoded area of a fruit in the image.

The original CenterNet uses heatmap to learn the foreground–background feature of the object with the following prediction and supervision methods: The input fruit image is denoted as *I* ∈ *R*^*W* × *H*^ (as shown in Fig. 5A, where *W* and *H* represent the width and height of the image, respectively). The heatmap branch predicts a feature map $\hat{H}\in {\left[0,,,1\right]}^{\frac{H}{r}\times \frac{W}{r}}$ to locate the center points of the objects in *I*. At the same time, the model encodes each object in *I* by using the Gaussian kernel generation formula [29] to form a ground truth heatmap $H\in {\left[0,,,1\right]}^{\frac{H}{r}\times \frac{W}{r}}$ (as in Fig. 5B, where *r* represents the downsampling factor) of the center points to supervise $\hat{H}$ pixel-to-pixel. In *H*, the coded value of the pixel point corresponding to the object’s center point is 1, and the value of the pixel point corresponding to the point further away from the center point follows the Gaussian distribution decreasing (as in Fig. 5C). When the coded value is less than the threshold [29], the coded value is set to 0. The coded value of the pixel point corresponding to the background is also set to 0. Therefore, the Gaussian nonzero coded value is distributed near the pixel point corresponding to the center of the object. Therefore, it can be seen that the method of *H* supervises $\hat{H}\;$in the heatmap branch reflects the model’s learning approach of foreground object features and image background features, which determines the model’s learning ability of the foreground–background features. This supervision method is implemented by designing the heatmap branch’s positive and negative sample allocation strategy.

**Fig. 6.**
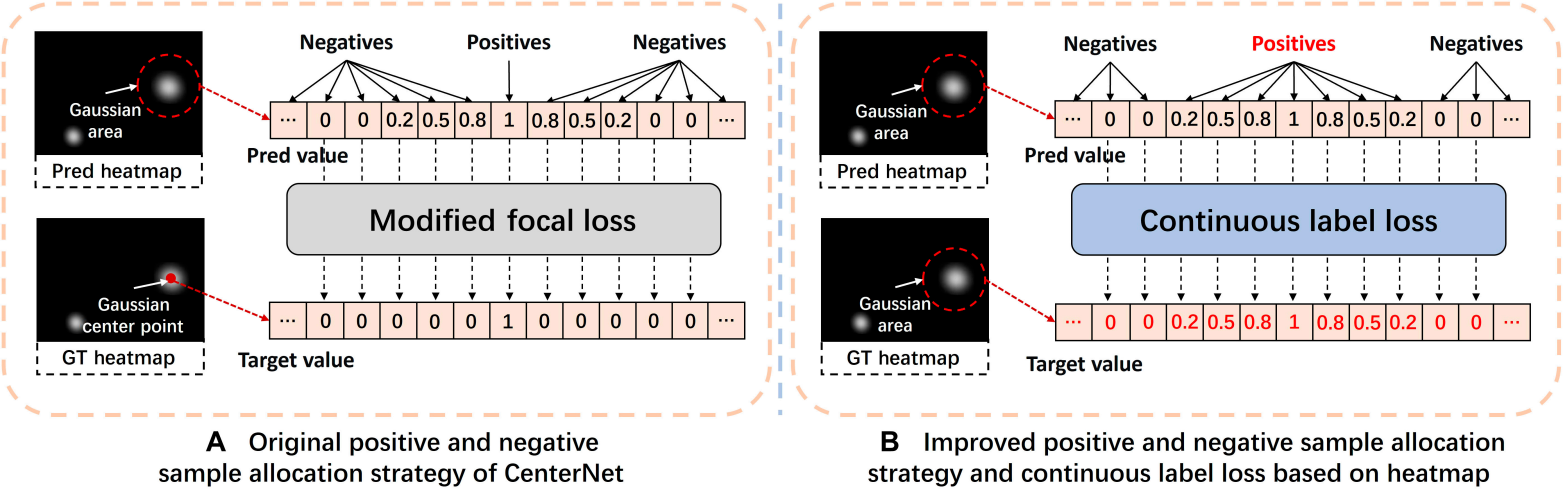
Comparison between (A) the original CenterNet positive and negative sample allocation strategy and (B) the improved method, Pred heatmap, GT heatmap, Positives, Negatives, Pred value, and Target value represent $\hat{H}$, *H*, positive sample, negative sample, prediction value, and supervision label value, respectively

In this paper, we improve a positive and negative sample allocation strategy of the heatmap branch and design a corresponding loss function, named the background domain-adaptive strategy based on sample allocation. The strategy includes 2 parts: the positive sample point expansion strategy based on heatmap and the continuous label value loss function. A comparison of the original strategy and the improved method is shown in Fig. 6.

*1. The positive sample point expansion strategy based on heatmap:* As shown in Fig. 6A, in the positive and negative sample allocation strategy of the heatmap branch of the original CenterNet, the sample points $\left(x,,,y\right)\in \hat{H}$ (with value ${\hat{H}}_{\mathrm{xy}}$) corresponding to the same pixel positions (*x*, *y*) ∈ *H* (with value *H_xy_* = 1, calculated by encoding the center points of the objects in the training image) are considered positive sample points of the branch. These points are supervised by a positive sample label with *value* = 1 to learn the foreground object feature. The remaining points in $\hat{H}$ are supervised by a negative sample label with *value* = 0 to learn the background feature of the image. In this case, the points with *H_xy_* = 1 are encoded only by the center of each fruit object, and the number of fruits in the image is much lower than the number of pixels in *H*; thus, there are far more points with *H_xy_* ≠ 1 than points with *H_xy_* = 1 in *H*. Therefore, the original CenterNet suffers from a severe imbalance problem between positive and negative samples. The number of positive sample points used for learning foreground features is extremely small and cannot focus on fruit foreground objects to address the problem of the background domain gap.

Therefore, to resolve the insufficient learning ability of the foreground feature of the model due to the imbalance of the original positive and negative sample allocation, we propose a positive sample point expansion strategy based on heatmap, as shown in Fig. 6B, which is described as follows: The sample points $(x,y)\in \hat{H}$ corresponding to the same pixel positions (*x*, *y*) ∈ *H* (with value *H_xy_* ≠ 0) are all considered positive sample points of the heatmap branch. These points are supervised by positive sample labels to learn the foreground features of the fruit objects. The sample points $(x,y)\in \hat{H}$ corresponding to the same pixel positions (*x*, *y*) ∈ *H* (with value *H_xy_* = 0) are considered negative sample points, supervised by negative sample labels to learn the background feature of the image. The improved sample allocation strategy algorithm is shown in Algorithm 1.
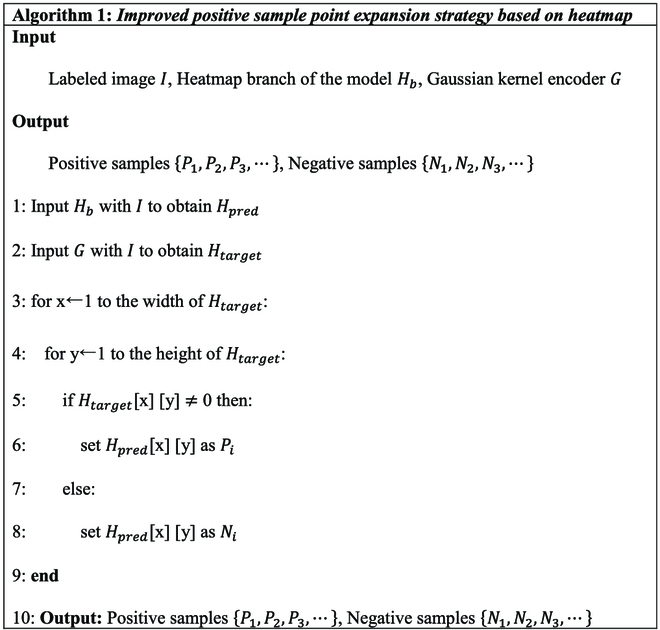


In the original positive and negative sample allocation, each fruit has one positive sample point. By applying the positive sample point expansion strategy based on heatmap in Algorithm 1, the number of positive sample points for each fruit becomes the number of nonzero points in the Gaussian distribution encoded by the fruit object. The number is determined by the size of the fruit object and the Gaussian threshold [29] in the encoding process, which is often greater than one. The above method dramatically expands the number of positive samples and can provide sufficient foreground training samples for the model, improving the ability of the model to extract the foreground feature of the fruit.

*2. The continuous label value loss function:* After formulating the rule for allocating positive and negative samples, the corresponding loss function must be designed to supervise them. The original CenterNet uses a modified focal loss function to supervise the heatmap branch, as shown in Eq. 3.

LH=−1N∑xy1−H^xyαlogH^xyifHxy=11−HxyβH^xyαlog1−H^xyotherwise (3)

In Eq. 3, N is the total number of pixel points in *H* or $\hat{H}$ (with the same resolution), ${\hat{H}}_{\mathrm{xy}}$ is the value of a sample point $(x,y)\in \hat{H}$ (predicted by the heatmap branch), and *H_xy_* is the value of the pixel point (*x*, *y*) ∈ *H* (ground truth heatmap) at the corresponding location, with default settings *α* = 2 and *β* = 4 [29]. The supervision label values of the loss function of the heatmap branch in the original CenterNet are discrete. The label *value* = 1 is used to supervise positive samples, and the label *value* = 0 is used to supervise negative samples. This supervision value setting is unsuitable for the improved positive and negative sample allocation strategy. Specifically, if the original loss function is used, since we consider all sample points in $\hat{H}$ that correspond to the same position in *H* with nonzero values as the positive samples, the newly added positive sample points are all supervised by the label *value* = 1. Therefore, the positive sample points from other positions have no difference from the positive sample points from the center points and have the same strength of supervision for model training. Thus, it is difficult for the model to locate the center point of the fruit object accurately. To address the above problem, we propose using a loss function [42] to make the label value continuous for the improved positive and negative sample allocation strategy. We refer to it as the continuous label value loss function, and its formula is shown in Eq. 4.

LH=−1N∑xyHxy−H^xyβ1−Hxylog1−H^xy+HxylogH^xy (4)

**Fig. 7.**
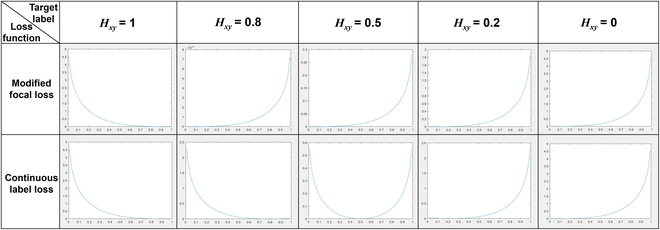
Comparison of the supervision effect between the original loss function (modified focal loss) and the continuous label value loss function, where the original loss function can be supervised only with 0 or 1, and the improved loss function can be supervised with any continuous value between 0 and 1.

In Eq. 4, the default setting β = 2 [42]. The label values of the continuous label value loss function are no longer limited to the supervision label *value* = 1 for positive samples and *value* = 0 for negative samples. The label can now be any decimal number between 0 and 1, and *H_xy_* can directly be used as the label value of the sample point $(x,y)\in \hat{H}$. For an intuitive representation of the loss function’s supervision effect, we show the loss values of the original loss function (modified focal loss function) and improved continuous label value loss function for various prediction values in Fig. 7.

In each panel of Fig. 7, the *x*-axis represents the prediction value ${\hat{H}}_{\mathrm{xy}}\in \left[0,,,1\right]$ of the sample point $(x,y)\in \hat{H}$, and the *y*-axis represents the loss value obtained when this point corresponds to the same position point (with values of *H_xy_* ∈ [0, 1]) in *H*. Examples of *H_xy_* in Fig. 7 are 1, 0.8, 0.5, 0.2, and 0. The analysis of each value of *H_xy_* is as follows.

1. When *H_xy_* = 1 (or *H_xy_* = 0), the corresponding same position point $(x,y)\in \hat{H}$ belongs to the sample point of the center point of the object (or the background). The loss values obtained by the continuous label value loss function and the modified focal loss function are consistent. That is, (*x*, *y*) is treated as a positive sample point (or negative sample point) for supervision.

2. When *H_xy_* is 0.8, 0.5, and 0.2, the corresponding same position points $(x,y)\in \hat{H}$ belong to the sample points closer, medium, and far away from the center point of the object, respectively. In the original modified focal loss function, (*x*, *y*) is supervised with a discrete value. Therefore, although the sample points corresponding closer to the center point are less strongly supervised, they are still supervised with a negative label *value* = 0, which cannot achieve the expansion of the positive sample points. Furthermore, if we change the partitioning condition of Eq. 3 to make (*x*, *y*) supervised by the loss function under the *H_xy_* = 1 condition, these newly added positive sample points are not distinguishable from the center positive sample points. In contrast, in the improved continuous label value loss function, (*x*, *y*) is supervised with a continuous value. In this way, (*x*, *y*) can be viewed as positive sample points to expand the positive samples, and new positive sample points can be directly supervised by different labels with *value* = *H_xy_*. Moreover, since *H_xy_* follows a Gaussian distribution encoded by the fruit object, the positive sample point corresponding to the center of the fruit receives stricter supervision than the nearby points. In addition, the remaining positive sample points are supervised with decreasing strength according to the Gaussian distribution as they get farther away from the center.

From this, we can distinguish between the center positive sample and the other positive samples by using the continuous label value loss function to the improved positive and negative sample allocation strategy. Therefore, the positive sample points at different positions have different impacts on the model update. This makes the supervision setting of newly added positive samples more reasonable.

In summary, by using the positive sample point expansion strategy based on heatmap and the continuous label value loss function, we can guide the training process to enhance the feature extraction capability of fruit foreground objects and can avoid interference under different background domains. The background domain-adaptive strategy based on sample allocation constructed through the above method can effectively bridge the domain gap of fruit background, thereby further improving the accuracy of pseudo-label generation.

## Results

The experiments in this paper verify the effectiveness of the proposed DomAda-FruitDet in improving the accuracy of the fruit auto labeling of the EasyDAM method [11,12]. The following introduces the datasets, evaluation metrics, experimental setup, and results.

### Datasets

To verify the effectiveness of DomAda-FruitDet, the dataset used comes from EasyDAMv1 [11] and EasyDAMv2 [12]. It includes 2 components: the source domain fruit dataset and the target domain fruit dataset. Orange is used as the source domain fruit, while apple, tomato, pitaya, and mango are used as the target domain fruits. There is a substantial domain gap between them, which is suitable for verifying the proposed model in this paper.

#### Source domain fruit dataset

**Fig. 8.**
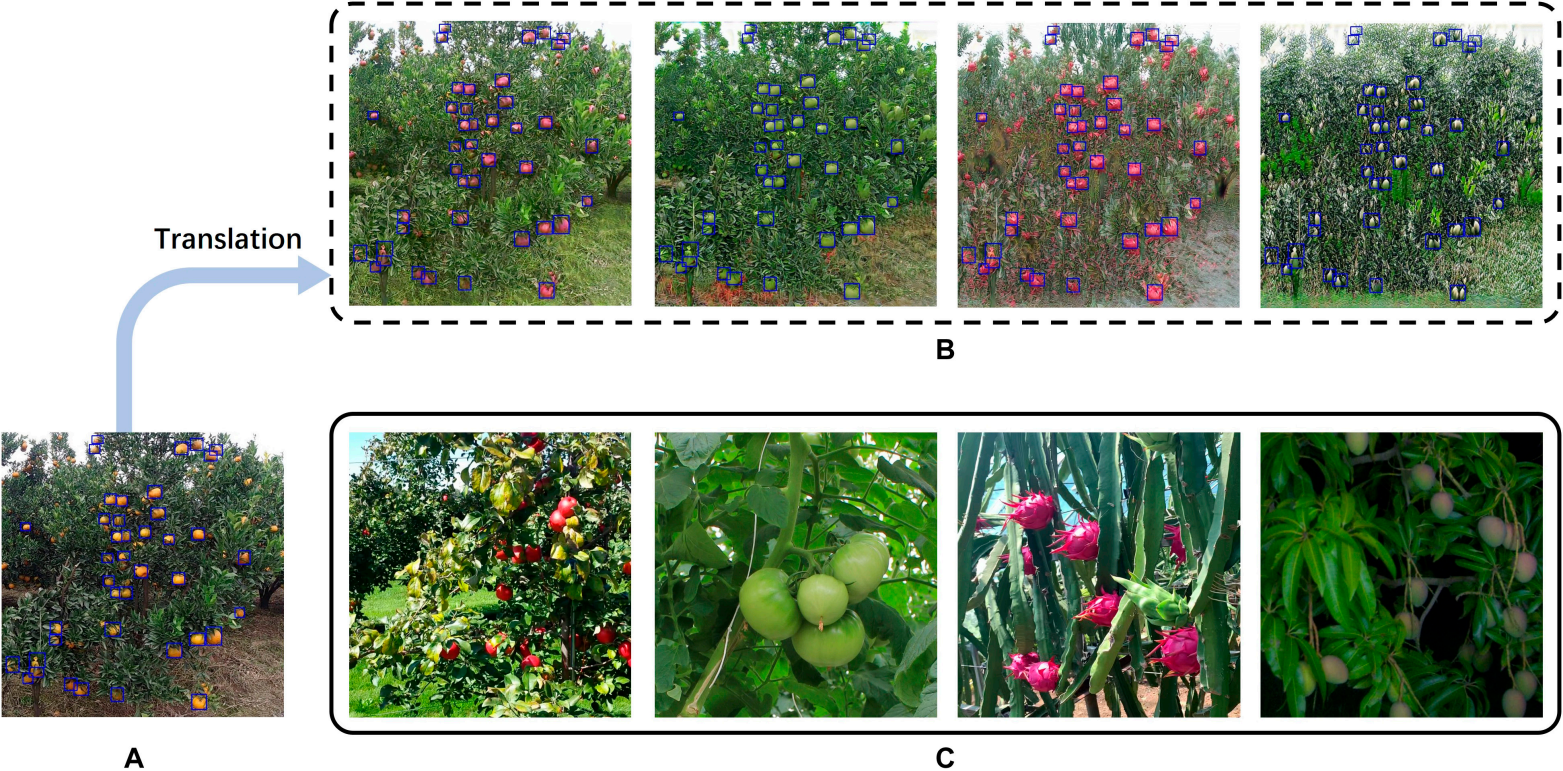
Example of the domain gap between source and target domain fruits (blue rectangular boxes in the image represent the annotation information of the “labeled image”). (A) Labeled source domain orange images; (B) various labeled target domain synthetic fruit images generated from the source domain; (C) various unlabeled target domain actual fruit images. The species in (B) and (C) are apple, tomato, pitaya, and mango, in the order from left to right. There is a huge domain gap between (B) and (C) in both the foreground and the background.

The source domain fruit dataset follows the orange dataset from the object detection datasets section of EasyDAMv1. It is mainly used as input for GANs to obtain the target domain synthetic fruit data for fruit detection model training. The orange images in this dataset were collected from an orchard in Sichuan Province, China, by using a DJI Osmo Action camera (DJI Innovation Technology Co., Ltd. in Shenzhen, China). A total of 664 orange images were collected and randomly divided into 464 training images and 200 testing images. The images were manually labeled by using relevant image annotation tools, as shown in Fig. 8A.

#### Target domain fruit dataset

The target domain fruit dataset consists of 2 parts: the target domain synthetic fruit dataset and the target domain actual fruit dataset. They are introduced below.

*1. Target domain synthetic fruit dataset:* The target domain synthetic fruit dataset includes 4 species: apple, tomato, pitaya, and mango. The apple and tomato come from the generated synthetic fruit in the image transformation module of EasyDAMv1. The pitaya and mango come from the generated synthetic fruit in the Fruit Image Translation Network Across-CycleGAN module of EasyDAMv2. These datasets are mainly used to train the fruit detection model. There are 464 synthetic fruit images for each species, as shown in Fig. 8B.

*2. Target domain actual fruit dataset:* The target domain actual fruit dataset also includes 4 species: apple, tomato, pitaya, and mango. The apple and tomato come from the object detection datasets section of EasyDAMv1. The pitaya and mango come from the fruit detection datasets section of EasyDAMv2. These datasets are mainly used to generate pseudo-label and to evaluate the fruit detection model. The specific description of each fruit is shown in Fig. 8C.

1. The apple dataset uses the publicly available dataset MinneApple [43]. There are 504 unlabeled images for the pseudo-label update method and 82 labeled images for the testing set of the detection model. The average pixel size of the apple in this dataset is 40 × 40, and it also contains some small objects (according to the Microsoft coco [44], the standard of the pixel area is less than 32 × 32, or according to the definition of the relative area, such as the object scale less than one-tenth of the original image), and with a complex shooting scene containing severe problems such as reflections, occlusion, and shadows. The high difficulty level of this dataset makes it suitable for application validation in our study.

2. The tomato dataset uses the publicly available dataset [45]. There are 598 unlabeled images for the pseudo-label update method and 150 labeled images for the testing set of the detection model. This dataset was taken at medium to close range, and when compared to that of the source domain orange fruit images obtained at longer distances, the tomato objects have a larger scale in the image. There is a degree of stacking, which makes it suitable for validation in our study.

3. The pitaya dataset was collected from an orchard in Beijing, China. A Samsung Galaxy S8 smartphone (Samsung Electronics Co., Ltd. in South Korea) was used as the data acquisition device, and additional images were collected from the internet and integrated into the dataset. There are 265 unlabeled images for the pseudo-label update method and 112 labeled images for the testing set of the detection model. Compared to that of the source domain orange, the scale of pitaya objects in this dataset spans a more extensive range, and there is a substantial difference between the lengths of the long and short axes of the pitaya. Therefore, it is necessary to generate bounding boxes that fit the objects better based on the pitaya morphology, which makes it suitable for validation in our study.

4. The mango dataset uses the publicly available dataset [46]. There are 598 unlabeled images for the pseudo-label update method and 104 labeled images for the testing set of the detection model. This dataset was collected under nighttime conditions. Compared to that of the source domain orange, there is a substantial background difference in the mango, and there are some stacking and dense growth problems. Therefore, it is suitable for application validation in our study.

### Experimental evaluation metrics

To verify the accuracy of the labels generated by using DomAda-FruitDet in the EasyDAM [11,12] method, the effectiveness of the proposed method was indirectly verified by the performance of the target domain fruit detection model obtained from the final training. The evaluation methods of the target domain fruit detection model use the precision, recall, F1 score, and average precision (AP) metrics. Higher values of the corresponding indexes indicate better detection model performance. Among them, the precision, recall, and F1 score are taken from the model performance balance point (i.e., where the precision value is approximately equal to the recall value), and the details of the evaluation methods can be found in previous work [11].

### Experimental setup

This experiment deployed a deep learning framework for model training and testing on a computer platform with an Intel Core i7-10700K central processing unit (64 GB of random access memory), GeForce RTX 3090 graphics processing unit (24 GB of video memory), and an operating system with ubuntu18.04LTS, using the Python 3.7 programming language to implement the construction, training, and validation of network models under the Pytorch 1.7.1 deep learning framework.

Model training: Set the batch size to 4 and the initial learning rate to 0.000125 for 100 training epochs. Decrease the learning rate by a factor of 10 at the 90th training epoch. Use Adam to optimize the overall objective.

### Experimental results

In this paper, we use DomAda-FruitDet as the fruit detection model for the EasyDAMv1 [11] and EasyDAMv2 [12] fruit auto labeling methods to address the problem of the domain gap and to further improve the accuracy of the label generation of the target domain actual fruit dataset. The comparison algorithm uses Orange-YOLO [13] (proposed in our team’s previous research) as the fruit detection model to generate the labels. Orange-YOLO achieved the best results thus far in EasyDAMv1 for the auto labeling of apple and tomato with a similar shape to the source domain orange and in EasyDAMv2 for the auto labeling of pitaya and mango with partial shape differences to the source domain orange. The 2 main parts of the experiment are as follows.

1. Based on the target domain synthetic fruit dataset to train the fruit detection model, the target domain pretrained apple, tomato, pitaya, and mango fruit detection models were constructed, denoted as *M_apple_*, *M_tomato_*, *M_pitaya_* and *M_mango_*, respectively (introduced in the “Comparative experiments on the effectiveness of the pretrained fruit detection model” section).

2. Based on the pretrained fruit detection models for the target domain, combined with the pseudo-label adaptive threshold selection strategy (a pseudo-label update method) in EasyDAMv2 [12], the pseudo-label of the unlabeled target domain actual apple, tomato, pitaya, and mango datasets was obtained and denoted as orange2apple, orange2tomato, orange2pitaya, and orange2mango experiments, respectively (introduced in the “Comparative experiments of label generation” section).

In addition, the ablation experiments of each module in DomAda-FruitDet are shown in the “Ablation experiments of DomAda-FruitDet” section.

#### Comparative experiments on the effectiveness of the pretrained fruit detection model

In this section, the labeled target domain synthetic fruit datasets are used as training data to construct the target domain pretrained models. Then, the unlabeled target domain actual fruit images are input to the pretrained models as testing data to verify the ability of the models to address the problem of the domain gap. In addition, to further compare the domain-adaptive detection capability of DomAda-FruitDet, we selected Single-DGOD [47], Every Pixel Matters [35], and SIGMA [48] of the domain-adaptive object detection methods for comparative experiments in this section. Single-DGOD cyclically extracts features from the training data via a cyclic-disentangled module. It improves the model’s domain adaptability under different application data distributions. The model is applied to the object detection of the Diverse-Weather Dataset of urban scene. Every Pixel Matters, a detection model based on adversarial feature learning, performs well in domain-adaptive detection. It designs the global and local classifiers to extract domain-invariant features to achieve street scene detection in different cities. Further, SIGMA improves the method of aligning domain features with graph matching, achieving vehicle–pedestrian detection under different conditions.

The comparison of the performance of the pretrained fruit detection models constructed by DomAda-FruitDet and others is shown in Table 1.

**Table 1.** Based on different detectors, comparative performance results of the target domain pretrained apple, tomato, pitaya, and mango fruit detection models

Experiment	Model	Precision	Recall	F1 score	AP
*M_apple_*	**Orange-Yolo**	**0.704**	**0.658**	**0.680**	**0.653**
	Single-DGOD	0.580	0.584	0.582	0.579
	Every Pixel Matters	0.659	0.662	0.661	0.677
	SIGMA	0.697	0.695	0.696	0.676
	**DomAda-FruitDet**	**0.744↑**	**0.744↑**	**0.744↑**	**0.773↑**
*M_tomato_*	**Orange-Yolo**	**0.723**	**0.725**	**0.724**	**0.711**
	Single-DGOD	0.391	0.400	0.395	0.317
	Every Pixel Matters	0.689	0.688	0.689	0.676
	SIGMA	0.713	0.698	0.705	0.717
	**DomAda-FruitDet**	**0.799↑**	**0.799↑**	**0.799↑**	**0.836↑**
*M_pitaya_*	**Orange-Yolo**	**0.710**	**0.710**	**0.710**	**0.728**
	Single-DGOD	0.595	0.596	0.596	0.594
	Every Pixel Matters	0.722	0.724	0.723	0.679
	SIGMA	0.686	0.683	0.685	0.689
	**DomAda-FruitDet**	**0.763↑**	**0.762↑**	**0.763↑**	**0.777↑**
*M_mango_*	**Orange-Yolo**	**0.678**	**0.678**	**0.678**	**0.687**
	Single-DGOD	0.420	0.425	0.423	0.439
	Every Pixel Matters	0.705	0.700	0.703	0.707
	SIGMA	0.716	0.710	0.713	0.711
	**DomAda-FruitDet**	**0.731↑**	**0.731↑**	**0.731↑**	**0.743↑**

**Fig. 9.**
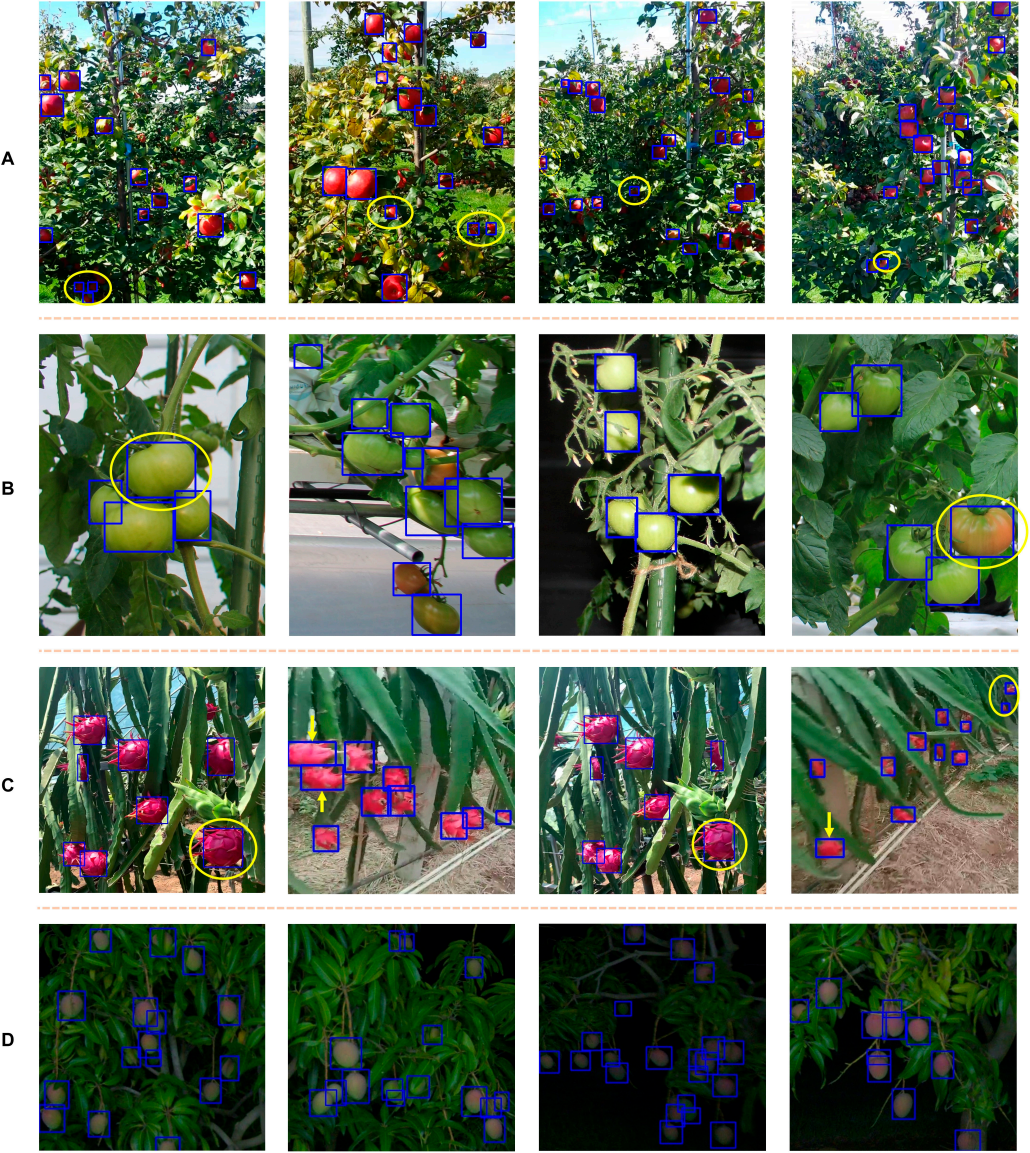
Visualization of the detection boxes for different species of fruit images in the actual orchard scene: (A to D) in the order of apple, tomato, pitaya, and mango. Where the blue rectangular box of the image represents the detection box, it can be converted into image label data to realize the auto labeling function of the fruit dataset. (A) Some apples are extremely small and can be detected, as shown in yellow circles. (B) The larger bounding boxes can be generated to enclose larger tomato objects, as shown in yellow circles. (C) The model can accurately detect pitayas of different scales, as shown in yellow circles, and generate bounding boxes that fit the elliptical shape of pitayas, as indicated by yellow arrows. (D) The model can accurately detect foreground objects in scenes with substantial background differences. The image shows the detection results obtained by the model trained with daytime mango images.

In the experiment of constructing the target domain pretrained fruit detection models, DomAda-FruitDet obtained AP values of 77.3%, 83.6%, 77.7%, and 74.3% in the apple, tomato, pitaya, and mango datasets, respectively, higher than the comparison algorithm. These results were higher than those of Orange-YOLO by 12.0%, 12.5%, 4.9%, and 5.6%, respectively, and the performance improvement was substantial. The visualization results when using DomAda-FruitDet to construct various pretrained fruit detection models and to detect various target domain actual fruits are shown in Fig. 9. With the domain gap, the performance improvements are analyzed as follows:

In the apple experiment, the target domain apple contains some extremely small objects with substantial scale differences from the source domain orange. The domain adaptive capability of DomAda-FruitDet for fruit scale characteristics enables the accurate detection of small apples in images. In the tomato experiment, the target domain tomato images were obtained by a camera at a closer distance. The tomatoes are at a larger scale, and there is a substantial difference in scale distribution between them and the source domain orange. DomAda-FruitDet is unaffected by the scale distribution of the source domain labeled boxes and can generate larger bounding boxes to cover the entire foreground area of the tomatoes. In the pitaya experiment, the target domain pitaya has an elliptical shape, while the source domain orange is nearly circular. DomAda-FruitDet can adaptively generate bounding boxes based on the shape and scale characteristics of pitayas. In the mango experiment, in addition to the scale difference between the target domain mango and the source domain orange, mango images were obtained in nighttime scenes. In contrast, the orange images were collected during the daytime. Therefore, there is a substantial difference in background between them. DomAda-FruitDet can accurately locate the foreground objects of mangoes under this domain gap.

The domain-adaptive detection capability of the pretrained fruit detection models constructed by DomAda-FruitDet can maximize the effectiveness of the subsequent pseudo-label update strategy. This capability provides a good foundation for achieving high-precision fruit auto labeling.

Meanwhile, with the same hardware devices in the “Experimental setup” section, we compare the parameters and the average inference speed of each model, and the results are shown in Table 2.

**Table 2.** Comparison of parameters and inference speed

Model	Parameters	FLOPs	FPS
Orange-YOLO	57M	184G	75
Single-DGOD	157M	37.5G	17
Every Pixel Matters	35M	83.6G	28
SIGMA	31M	78.4G	33
DomAda-FruitDet (proposed)	16M	56.6G	54

As can be seen, the inference speed of DomAda-FruitDet is higher than others but lower than Orange-YOLO. However, accuracy is the primary evaluation metric in auto labeling tasks, and the importance is priority over speed. The inference speed of 54 FPS can already fully satisfy the practical application requirements.

#### Comparative experiments of label generation

In this section, based on the pretrained fruit detection model for the target domain constructed in the “Comparative experiments on the effectiveness of the pretrained fruit detection model” section, the unlabeled target domain actual fruit images are input to the pretrained model for detection and output in the form of pseudo-label data. The labels are then optimized, and the fruit detection model is updated by the pseudo-label adaptive threshold selection strategy. The performance of the updated model indirectly verifies the accuracy of the final label generation for the target domain actual fruit dataset under the domain gap. We compared the label generation accuracy of DomAda-FruitDet and Orange-YOLO for the EasyDAM method, as shown in Table 3.

**Table 3.** Comparison of label generation accuracy for target domain apple, tomato, pitaya, and mango based on different fruit detection models

Experiment	Model	Precision	Recall	F1 score	AP
*orange*2*apple*	Orange-Yolo	0.836	0.834	0.835	0.897
	**DomAda-FruitDet**	**0.841↑**	**0.842↑**	**0.842↑**	**0.909↑**
*orange*2*tomato*	Orange-Yolo	0.790	0.786	0.788	0.823
	**DomAda-FruitDet**	**0.853↑**	**0.860↑**	**0.857↑**	**0.908↑**
*orange*2*pitaya*	Orange-Yolo	0.782	0.778	0.780	0.821
	**DomAda-FruitDet**	**0.853↑**	**0.853↑**	**0.853↑**	**0.883↑**
*orange*2*mango*	Orange-Yolo	0.818	0.816	0.817	0.850
	**DomAda-FruitDet**	**0.894↑**	**0.894↑**	**0.894↑**	**0.940↑**

In the fruit label generation experiment, DomAda-FruitDet obtained AP values for the target domain actual apple, tomato, pitaya, and mango datasets, which were 90.9%, 90.8%, 88.3%, and 94.0%, respectively. The F1 score values were 84.2%, 85.7%, 85.3%, and 89.4%, respectively. Compared with those of Orange-YOLO, the AP values were improved by 1.2%, 8.5%, 6.2%, and 9.0%, respectively. Various pretrained fruit detection models based on DomAda-FruitDet can maximize the effectiveness of the pseudo-label adaptive threshold selection strategy, further obtaining detection models with higher generalization. At the same time, in the final label generation results, DomAda-FruitDet can obtain high-precision fruit labels under the domain gap. The AP values of apple, tomato, and mango can reach more than 90%, ensuring the labeling quality of the final constructed fruit datasets of various target domains.

#### Ablation experiments of DomAda-FruitDet

To further validate the effectiveness, we compared the various improved strategies in DomAda-FruitDet, including the foreground domain-adaptive structure based on double prediction layers (denoted as DPL) and the background domain-adaptive strategy based on sample allocation (denoted as SA). The ablation experiments of the pretrained detection model and the label generation are shown in Tables 4 and 5 (where CenterNet + DPL + SA is the DomAda-FruitDet).

**Table 4.** Ablation experiments of the pretrained detection model

Experiment	Model	Precision	Recall	F1 score	AP
*M_apple_*	CenterNet	0.688	0.688	0.688	0.683
	CenterNet+DPL	0.727	0.727	0.727	0.753
	CenterNet+SA	0.705	0.705	0.705	0.730
	CenterNet+DPL+SA	**0.744↑**	**0.744↑**	**0.744↑**	**0.773↑**
*M_tomato_*	CenterNet	0.740	0.736	0.738	0.777
	CenterNet+DPL	0.795	0.795	0.795	0.829
	CenterNet+SA	0.774	0.792	0.783	0.815
	CenterNet+DPL+SA	**0.799↑**	**0.799↑**	**0.799↑**	**0.836↑**
*M_pitaya_*	CenterNet	0.717	0.717	0.717	0.728
	CenterNet+DPL	0.747	0.739	0.743	0.751
	CenterNet+SA	0.733	0.729	0.731	0.740
	CenterNet+DPL+SA	**0.763↑**	**0.762↑**	**0.763↑**	**0.777↑**
*M_mango_*	CenterNet	0.707	0.707	0.707	0.716
	CenterNet+DPL	0.727	0.727	0.727	0.722
	CenterNet+SA	0.715	0.724	0.720	0.735
	CenterNet+DPL+SA	**0.731↑**	**0.731↑**	**0.731↑**	**0.743↑**

**Table 5.** Ablation experiments of the label generation

Experiment	Model	Precision	Recall	F1 score	AP
*orange*2*apple*	CenterNet	0.799	0.799	0.799	0.837
	CenterNet+DPL	0.828	0.827	0.828	0.890
	CenterNet+SA	0.809	0.809	0.809	0.868
	CenterNet+DPL+SA	**0.841↑**	**0.842↑**	**0.842↑**	**0.909↑**
*orange*2*tomato*	CenterNet	0.793	0.792	0.793	0.833
	CenterNet+DPL	0.835	0.836	0.836	0.879
	CenterNet+SA	0.820	0.821	0.821	0.859
	CenterNet+DPL+SA	**0.853↑**	**0.860↑**	**0.857↑**	**0.908↑**
*orange*2*pitaya*	CenterNet	0.775	0.772	0.774	0.775
	CenterNet+DPL	0.849	0.849	0.849	0.864
	CenterNet+SA	0.791	0.791	0.791	0.810
	CenterNet+DPL+SA	**0.853↑**	**0.853↑**	**0.853↑**	**0.883↑**
*orange*2*mango*	CenterNet	0.830	0.830	0.830	0.844
	CenterNet+DPL	0.885	0.885	0.885	0.926
	CenterNet+SA	0.833	0.833	0.833	0.875
	CenterNet+DPL+SA	**0.894↑**	**0.894↑**	**0.894↑**	**0.940↑**

As can be seen from the ablation experiments, with the foreground domain-adaptive structure based on double prediction layers and the background domain-adaptive strategy based on sample allocation, we obtain higher accuracy based on the baseline model. The model detection effect reaches the best when the two are combined.

## Discussion

In this paper, we propose a cross-domain detection model, DomAda-FruitDet, which can be adapted to fruit morphology to generate detection boxes and to address the problem of the domain gap in the EasyDAM [11,12] method. The model is highly generalizable when there is an incomplete match of fruit characteristics between supervisory information and application data. Our approach can reduce the effects of factors such as shooting angle, shooting distance, and environmental changes. DomAda-FruitDet is further compared with Orange-YOLO [13] to demonstrate the effectiveness of the model. Meanwhile, we can find from the fruit label generation experiments that the AP of pitaya failed to reach more than 90%, which is less accurate than other fruits. This is because some of the pitayas are heavily obscured by vines (with thick branches), and only a small area of the fruit is visible, making it difficult for the model to locate such objects. In addition, a certain degree of occlusion in other fruits also affects label generation accuracy. In a recent study, we noticed some work [49,50] to improve detection performance by setting up attention mechanisms in the model for the fruit occlusion problem. We plan to subsequently analyze this idea and to conduct further research to improve the effectiveness of fruit auto labeling.

In summary, the model proposed in this paper for fruit auto labeling in smart orchards can effectively generate the required labeled fruit dataset and can improve the efficiency of orchard intelligence. In addition, as a complete object detection model, it can be used for other detection tasks. With domain adaptive detection performance, it can be applied to tasks with a domain gap, such as vehicle and pedestrian detection in different street scenes and detection tasks where the synthetic model is training data and the actual object is application data.

## Data Availability

The data and executable files used in this paper will be available upon request at: https://github.com/I3-Laboratory/DomAda-FruitDet_dataset.
